# Supplementary material for: National representative analysis of unilateral hearing loss and hearing aid usage in South Korea
Source: PLoS One. 2020 Apr 23;15(4):e0232112. doi: 10.1371/journal.pone.0232112 (PMC7179862; doi:10.1371/journal.pone.0232112)
Supplement: Attachment — Submitted filename: Response_letter_final.docx [file pone.0232112.s001.docx]

February 10, 2020

Joerg Heber

Editor

PLOS ONE

Dear Dr Heber,

Please consider our revised manuscript, “National representative analysis of unilateral hearing loss and hearing aid usage in South Korea”, for publication in *PLOS One*

We appreciate the interest that the editors and reviewers have taken in our manuscript and the constructive criticism they have given. We have addressed the major concerns of the reviewers. We have also included a point-by-point response to the reviewers in addition to making the changes in the manuscript. Changes to the text in the manuscript are marked in red. Our main findings remain unchanged.

This manuscript has not been published or presented elsewhere in part or in entirety and is not under consideration by another journal. The study design was approved by the appropriate ethics review board. We have read and understood your journal’s policies, and we believe that neither the manuscript nor the study violates any of these. There are no conflicts of interest to declare.

Bo Gyung Kim, MD, PhD

Department of Otorhinolaryngology-Head and Neck Surgery, Soonchunhyang University College of Medicine

Address: 170 Jomaru-ro, Wonmi-gu, Bucheon 14584, Korea

Tel: +82-32-621-6951

Fax: +82-32-621-5018

E-mail: bgkim@schmc.ac.kr

5. Review Comments to the Author

Please use the space provided to explain your answers to the questions above. You may also include additional comments for the author, including concerns about dual publication, research ethics, or publication ethics. (Please upload your review as an attachment if it exceeds 20,000 characters)

Reviewer #1: 1. REVIEWER SUMMARY: This is a report of unilateral hearing loss prevalence and also hearing aid use in South Korea. It uses the KNHANES, which should be representative of the actual population through sample weighting. Reporting of accurate, unbiased prevalence statistics is very important. While this has been done, for example, in the US, it is important to replicate in other countries because prevalence may differ. This study contains these important data, but there are a number of limitations detailed below. For example, multivariable regression was used but there was no obvious mention of what the variables were that were adjusted for in the model, which is critical information. In addition, the definition of unilateral hearing used a 40 dB cutoff, which is different from other papers that were mentioned in the discussion. While the authors are free to chose whatever defintion they wish, they should (a) justify the definition and (b) not make comparisons to studies that use a different definition unless they explicitly discuss this.

2. UNIQUE ASPECTS: There have been a few studies on hearing loss prevalence using the KNHANES that were not mentioned.

For example: <https://www.ncbi.nlm.nih.gov/pubmed/25216153>

and <https://www.ncbi.nlm.nih.gov/pubmed/28196098>.

Both, in fact, reported unilateral hearing loss, although different definitions may have been used. This should be mentioned.

3. STUDY DESIGN: Cross-sectional

4. WRITING STYLE: Mostly understandable English. Abstract needs proofreading by a native English speaker. Many wording issues, mostly in the abstract:

Line 37-38. What does "occupation presence" mean. Does that mean having an occupation (i.e., "working")?

→We would first like to thank reviewer 1 for their constructive criticism and suggestion. The term “occupation presence” stands for having an occupation, as you correctly assumed. We have changed “occupation presence” to “occupation status” so that it conveys our meaning better to the readers.

Line 34. Do not begin a sentence with And.

→. We have revised the text according to your recommendations.

Lines 40-42 "In comparison of hearing aid users and non-users, noise exposure during work in hearing aid users was significantly lower than hearing aid non-users." Needs rewording.

→ We have revised our text according to your suggestion. It now reads;

*“Prevalence of noise exposure at work in hearing aid users was significantly lower than the prevalence of noise exposure at work in users with no hearing aid.”*

Lines 42-43. "The prevalence of hearing aid use in patients with unilateral hearing loss in South Korea is very low compared as other countries." Do you mean that the low use is similar to other countries? In that case, you want to write "comparable to". Otherwise it should be "compared with" or "compared to".

→ We have revised the text according to your suggestions.

Line 43-45. "Public health education is needed for increased insight and auditory rehabilitation for unilateral hearing loss should be recommended actively." Needs rewording.

→Thank you for your comment. We have changed the text and it now reads as:

*“Public health education is needed in order to increase public awareness of unilateral hearing loss, hearing aid adoption and its continued usage. Auditory rehabilitation should be actively recommended to patients with unilateral hearing loss.”*

Lines 104-106. "To determine the prevalence of tympanic membrane perforation and cholesteatoma, including retraction pocket and otitis media with effusion" Needs rewording. Retraction pockets and otitis media with effusion are not types of tympanic membrane perforations or cholesteatoma.

→Thank you for your comment. We have the text and it now reads as:

*“To determine the prevalence of tympanic membrane perforation and cholesteatoma, as well as the retraction pocket and otitis media with effusion,”*

5. SPECIFIC COMMENTS:

Big data is sort of a buzz word. This is a relatively big dataset, but there are many studies that use KNHANES or the US NHANES and they normally don't use the term big data, I would at least remove from the title. You could maybe replace with something like "Nationally representative" to indicate it wasn't just based on a single hospital's audiology clinic.

→ We agree with your concern. We have changed the title as you recommended to “National representative analysis of unilateral hearing loss and hearing aid usage in South Korea”

Line 26-27. Do you mean lacking in South Korea? There are other studies using population-level data, e.g., reference #13.

→ Of course, several studies have been published, such as reference # 13, however, there are very few studies on the use of UHL and hearing aids in Korea as well as in other countries, compared to studies on bilateral SNHL.

Line 48-51. You state the incidence of unilateral hearing loss and then in the next sentence state that the true incidence is not known. In between, you should explain the limitations of the existing methods, i.e., why they are not accurate.

→ As we mentioned earlier, there are very few studies on the use of UHL and hearing aids in South Korea.

Line 57. It is Purcell, not Percell.

→. We have revised our text accordingly.

Line 113-115. Your definition of PTA >= 41 dB for hearing loss is different from many of the recent American epidemiology papers which use >=26 dB. As I am familiar, 25-40 dB is often considered mild hearing loss, which is still hearing loss. How you define hearing loss is a matter of debate and certainly varies. I would place your definition of unilateral hearing loss in the abstract to make this clear (i.e., >= 40 dB PTA at 0.5, 1, 2, 3 KHz in the worse hearing ear). I would also place this definition as a footnote in all of your tables.

→Thank you for your valuable comment and suggestion.

Many studies define unilateral hearing loss as PTA >= 26 dB. However, the KNHANES defined unilateral hearing loss as PTA >=41 dB. Moreover, the prevalence of unilateral hearing loss in our study is much lower than other studies, which define unilateral hearing loss as PTA >=25 dB. If we change the definition of PTA >=26dB rigidly, there would be no one who used a hearing aid.

I would recommend not double-spacing the tables so they are not spread across four pages (i.e., Table 3).

→Thank you for your comment. We have revised the tables as you suggested.

Table 1. You present categories, but the total data. It would be helpful to have an overall column that is not divided into a severity category. i.e, >=41 dB. It would also be helpful to have an age >= 12 row to represent "any age."

→We have added the overall column in Table 1 as you recommended.

Line 121. It seems you presented the prevalence of UHL with standard eror, not 95% CI (e.g., tables 1, 2, 4)

→ We agree with your comment and now have presented the prevalence of UHL with a standard error. We have changed the text in Methods:

*The prevalence of UHL was calculated and reported as a weighted percentage with standard error.*

Line 170. "In multivariable logistic regression..." You have to state what the other variables that you adjusted for were! This should also be clear in Table 3, e.g., in a footnote. This should also be in the methods.

→ The independent variables of the multivariable logistic regression model are the occupational status, the hearing threshold in the better ear, and the hearing threshold in the worse ear. There are no calibration parameters other than those shown in the table. In the univariable analysis results, the significant variables were considered as independent variables of the multivariable analysis. Among the five variables that were significant in univariable analysis, otitis media and dizziness were difficult to estimate due to an insufficient number of patients.

The Table title and results text (and methods) should all match and reflect what was actually done. E.g., Table 3 title states the outcome is hearing aid adoption or cochlear implantation. The results and methods text do not mention cochlear implantation. In fact, nowhere else in the paper is cochlear implant mentioned as far as I see.

→ We have removed the term “cochlear implantation” from the title for Table 3.

I am very confused by the different method used to assess factors associated with hearing aid use versus factors associated with hearing aid adoption. Hearing aid use and hearing aid adoption are VERY similar, with the later also including people who have a hearing aid but rarely use them. Why do you use regression to look for variables associated with hearing aid adoption, but then you use T-tests or Chi-squared tests for variables associated with hearing aid use.

→ We agree with your concern. Hearing aid use and hearing aid adoption are very similar, however, the prevalence of using hearing aids here refers to good compliance/usage. We analyzed the variables affecting hearing aid adoption as well as its use. We used regression analysis with hearing aid use but the results of the analysis were unreliable due to the small number of the data according to the independent variables. Therefore, we analyzed the variables using the T-test or Chi-squared tests instead of logistic regression.

Lines 193-197. This is very important. If you are comparing your findings of unilateral hearing loss to other papers measuring hearing loss, your definitions must be the same! You cite ref #16 Agrawal but she used >25 dB to define hearing loss, but you used >40 dB. Those will lead to VERY different numbers! Also ref #13 Golub et al used > 25 dB. I think ref #17 Chia et al also used >25 dB.

→ As mentioned earlier, the KNHANES defined unilateral hearing loss as PTA >=41 dB. Moreover, the prevalence of unilateral hearing loss in our study is much lower than other studies, which define unilateral hearing loss as PTA >=25 dB. If we change the definition of PTA >=26dB rigidly, there would be no one who used a hearing aid. The criteria are less strict than other studies, but with lower prevalence, we believe our results are meaningful.

The similarity of the terms hearing aid use and hearing aid adoption is a little confusing. In the results and the discussion, I would clarify that use means something like current active use. And adoption means something like current active or rare use.

→ We have clarified the definition as you suggested in the Methods.

Methods - you must define how all variables were measured. This includes all variables in the table. For example, how was noise exposure during work measured and what does it even mean? It can mean extremely loud noise such as explosions or factory noise. This could injure your hearing. It can also mean background noise like people talking. This would not injure your hearing but can make hearing with a mild hearing loss more difficult.

→ We used the data obtained from the 2009-2012 KNHANES. Participants were asked for the presence of noise but there was no query on the degree of noise.

Line 249 "In conclusion, the prevalence of hearing aid use in patients with UHL is very low in South Korea compared to other countries." This is your key concluding statement, but I am not sure I can trust it because your definition of UHL may be different from the studies you compared to (see above)

→ The criteria are less strict than other studies but with a lower prevalence, we think our results are meaningful. We have further commented that the criteria are different from other cited studies, in the limitations.

Reviewer #2:

 Line 8; This article did not focus on congenital UHL. So, introduction like this is inappropriate.

→We would like to thank reviewer #2 for their insightful comments and suggestions. Our topic is on unilateral hearing loss and we do not focus on congenital UHL. However, we wanted to mention the effects of UHL in both, children and adults.

 Line 63; What does “development” mean? Development of what?

→ “Development” here refers to the developing ability to solve tasks such as language, communication, socialization, and motor skills.

 Line 100; Why did you exclude children younger than 12 years-old?

→ We used the data obtained from the 2009-2012 KNHANES. Pure tone audiometry was conducted in participants over 12 years old. We, therefore, excluded children younger than 12 years old.

 Line 113-4; Was this definition of PTA suitable for UHL or asymmetric HL? General criteria for “hearing loss” is 26dBHL. 40dBHL is used as the criteria of “disabling hearing loss”, NOT “hearing loss” for adults. The criteria of “disabling hearing loss” is 30dBHL for children. Please, provide the reference of authors’ criteria for UHL. I think that authors should have used other term rather than UHL if they insisted on the 40dBHL-criteria.

→Thank you for your valuable comment and suggestion.

Many studies define unilateral hearing loss as PTA >= 26 dB. However, the KNHANES defined unilateral hearing loss as PTA >=41 dB. Moreover, the prevalence of unilateral hearing loss in our study is much lower than other studies, which define unilateral hearing loss as PTA >=25 dB. If we change the definition of PTA >=26dB rigidly, there would be no one who used a hearing aid. The criteria are less strict than other studies, but with lower prevalence, we believe our results are meaningful.

 Line 142-3; Authors told that they analyzed the hearing use in people with UHL, but their data concluded the hearing aid was more used in asymmetric HL. How do you think about my point-out?

→We fully agree with your comments. It is believed that patients with unilateral SNHL have a decreased need for hearing aids and it is not necessary to wear hearing aids, even when in noisy situations. People with asymmetric HL tend to need hearing aids more than UHL.

 To identify the factors associated with hearing aid adoption, logistic regression analyses were performed. But to identify factors associated with hearing aid use, t-tests and Chi-square tests were performed. Why did you use different statistical methods?

→ Hearing aid use and hearing aid adoption are very similar, as you commented, however, the prevalence of using hearing aids here refers to good compliance/usage. We analyzed the variables affecting the hearing aid adoption as well as its usage. We used regression analysis with hearing aid use but the results of the analysis appeared unreliable due to the small number of the data according to the independent variables. Thus, we analyzed the variables using the T-test or Chi-squared tests instead of logistic regression.

 Table 1; What was the “weighted percentage”? On what was it weighted?

→ We have expanded on the weighted percentage in the Statistical analysis:

*To reflect the national population estimates, sample weights were applied in all analyses.*

 Table 2; Why was PTA average of better ear classified into {≤ 20dB}, {> 20dB, ≤ 30 dB} and {> 30 dB, ≤ 40 dB}?

→ Generally, it can be considered significant when the difference is about 10dB, therefore, we classified it in Table 2.

 Table 2; please, rewrite the data correctly.

→ Thank you for your comments. We have rewritten the data in Table 2.

 Table 3; Cochlear implantation? What is this?

→Thank you for your comment. We have removed the term “cochlear implantation” from the title of Table 3.
