# Supplementary material for: National representative analysis of unilateral hearing loss and hearing aid usage in South Korea
Source: PLoS One. 2020 Apr 23;15(4):e0232112. doi: 10.1371/journal.pone.0232112 (PMC7179862; doi:10.1371/journal.pone.0232112)
Supplement: Attachment — Submitted filename: 2ndrevision_responseletter.docx [file pone.0232112.s002.docx]

Reviewer #1: Thank you for the revisions and the detailed responses to my comments. Several of my comments from the prior submission remain unaddressed in the manuscript. In general, when a reviewer has a comment that requires a clarification, they would like the clarification to be inside the manuscript, not just in the response to reviewer comments. If the reviewer had a question, then many readers may have the same question.

1. NEW COMMENTS. (Line numbers refer to the new submission version with tracked changes)

Line 42 "...noise exposure at work in users with no hearing aid" should be "...noise exposure at work in those with no hearing aid"

> Response: Thank you for your careful review. We have revised our text accordingly. It now reads:

*“The prevalence of noise exposure at work in hearing aid users was significantly lower than the prevalence of noise exposure at work in those with no hearing aid.”* (revised lines 42-44)

2. PRIOR COMMENTS that were not addressed by modifications to the manuscript. NOTE that line numbers here refer to the **first submission**) Your comments are denoted by >>. My new comments are denoted by >>>

Line 26-27. Do you mean lacking in South Korea? There are other studies using population- level data, e.g., reference #13.

>>Of course, several studies have been published, such as reference # 13, however, there are very few studies on the use of UHL and hearing aids in Korea as well as in other countries, compared to studies on bilateral SNHL.

>>>Thank you, I understand. Please make this more clear in the manuscript by rewording the first sentence of the abstract to add "in Korea" to the end of this current sentence: "A definitive study on the prevalence of adult unilateral hearing loss and hearing aid rehabilitation is lacking."

> Response: Thank you for your comment. We have edited the text for clarity and it now reads:

*“A definitive study on the prevalence of adult unilateral hearing loss and hearing aid rehabilitation is lacking in Korea.*

Line 113-115. Your definition of PTA >= 41 dB for hearing loss is different from many of the recent American epidemiology papers which use >=26 dB. As I am familiar, 25-40 dB is often considered mild hearing loss, which is still hearing loss. How you define hearing loss is a matter of debate and certainly varies. I would place your definition of unilateral hearing loss in the abstract to make this clear (i.e., >= 40 dB PTA at 0.5, 1, 2, 3 KHz in the worse hearing ear). I would also place this definition as a footnote in all of your tables.

>>Thank you for your valuable comment and suggestion. Many studies define unilateral hearing loss as PTA >= 26 dB. However, the KNHANES defined unilateral hearing loss as PTA >=41 dB. Moreover, the prevalence of unilateral hearing loss in our study is much lower than other studies, which define unilateral hearing loss as PTA >=25 dB. If we change the definition of PTA >=26dB rigidly, there would be no one who used a hearing aid.

>>>Thank you for the explanation. You justify why you use >=40 dB. I understand this. However, because this is not the worldwide standard definition of hearing loss, I recommend doing this (repeated from above): Place your definition of unilateral hearing loss in the abstract to make this clear (i.e., >= 40 dB PTA at 0.5, 1, 2, 3 KHz in the worse hearing ear). I would also place this definition as a footnote in all of your tables."

>Response: Thank you for clarifying and we agree. We have added the definition of unilateral hearing loss in the abstract and all tables as you suggested.

*Unilateral hearing loss was defined as pure tone average ≥ 41 dB in the worse hearing ear, and < 41 dB in the other ear assessed at 0.5, 1.0, 2.0, and 3.0 kHz.* (revised lines 34-36)

Line 170. "In multivariable logistic regression..." You have to state what the other variables that you adjusted for were! This should also be clear in Table 3, e.g., in a footnote. This should also be in the methods.

>>The independent variables of the multivariable logistic regression model are the occupational status, the hearing threshold in the better ear, and the hearing threshold in the worse ear. There are no calibration parameters other than those shown in the table. In the univariable analysis results, the significant variables were considered as independent variables of the multivariable analysis. Among the five variables that were significant in univariable analysis, otitis media and dizziness were difficult to estimate due to an insufficient number of patients.

>>>I understand. Often in multivariable regression, there are other variables that are non-significant (i.e., pontential confounders). Since all 3 variables you describe are significant, it is not clear that you did not include any other variables in the model. To make this clear, at the end of the sentence "In multiple logistic regression..." please insert another sentence and explain that there were no other variable present in the multivariable model. Please also, like I said, put this information in the methods and in a Table 3 footnote.

> Response: Thank you for your comment. We have revised our text according to your suggestion. It now reads:

*“In multivariable logistic regression analyses occupational status (OR 3.750, 95% CI 1.441–9.763), hearing threshold in the better ear (OR 1.088, 95% CI 1.029–1.151), and hearing threshold in the worse ear (OR 1.031, 95% CI 1.005–1.058) were significantly associated with hearing aid adoption (Table 3). There were no calibration parameters other than those shown in the table.”*

Lines 193-197. This is very important. If you are comparing your findings of unilateral hearing loss to other papers measuring hearing loss, your definitions must be the same! You cite ref #16 Agrawal but she used >25 dB to define hearing loss, but you used >40 dB. Those will lead to VERY different numbers! Also ref #13 Golub et al used > 25 dB. I think ref #17 Chia et al also used >25 dB.

>>As mentioned earlier, the KNHANES defined unilateral hearing loss as PTA >=41 dB. Moreover, the prevalence of unilateral hearing loss in our study is much lower than other studies, which define unilateral hearing loss as PTA >=25 dB. If we change the definition of PTA >=26dB rigidly, there would be no one who used a hearing aid. The criteria are less strict than other studies, but with lower prevalence, we believe our results are meaningful.

>>>Thank you and I understand why you used >40 dB: you had no choice because this is what KHANES used. However, please modify the text here to explain that your definition of hearing loss is different from the papers you are comparing it to. The following statement is completely misleading without an explanation: "In the present study conducted in South Korea, the prevalence of UHL determined via KNHANES data was 4.91%. This is lower than the prevalence of 7.9%–13.3% in the general population [16, 17] and the prevalence of UHL was 7.2% in the US [13]." You must insert a sentence here to say like: "However, our definition of hearing loss was >40 dB which is different from something the previously cited studies, which used >25 dB to define hearing loss. Thus we can not make direct comparisons."

> Response: Thank you for your valuable comment and suggestion. For clarity, we have added the sentences as you recommended. (revised lines 201-203)

Methods - you must define how all variables were measured. This includes all variables in the table. For example, how was noise exposure during work measured and what does it even mean? It can mean extremely loud noise such as explosions or factory noise. This could injure your hearing. It can also mean background noise like people talking. This would not injure your hearing but can make hearing with a mild hearing loss more difficult.

>>We used the data obtained from the 2009-2012 KNHANES. Participants were asked for the presence of noise but there was no query on the degree of noise.

>>>Thank you. Please address my comment. Please define how all the variables were measured or defined, briefly according to the KHANES manual, in the methods. If there is no room, place this in a supplement or a reference to the KHANES manual.

>Response: Thank you for your comment. We have added the KHANES manual as reference #15.
